# Mindset as a resilience resource and perceived wellness of first responders in a South African context

**DOI:** 10.4102/jamba.v14i1.1312

**Published:** 2022-06-30

**Authors:** John W. O’Neil, Leandri Kruger

**Affiliations:** 1 African Centre for Disaster Studies, Faculty of Natural and Agricultural Sciences, North-West University, Potchefstroom, South Africa; 2 SA Army Assessment Centre, Military Psychological Institute, South African National Defence Force, Pretoria, South Africa; 3 Unit for Environmental Sciences and Management, African Centre For Disaster Studies, Faculty of Natural and Agricultural Sciences, North-West University, Potchefstroom, South Africa

**Keywords:** mindset, perceived wellness, resilience, resilience resources, first responders

## Abstract

The global increase in frequency and intensity of disasters and emergency situations has a major disruptive effect on societies that is especially visible in Africa, where conflict, poverty, diseases and social unrest are some of the biggest factors contributing to societal vulnerability. Developing countries such as South Africa are vulnerable to the impact of disaster situations that strain the society’s ability to deal with these emergencies. First responders play an important function responding to disasters but are exposed to work-related stressors that could impact their performance. Several international studies make a link between wellness, performance and resilience and the use of resilience resources in the development and enhancement of wellness, indicating that resilience resources such as a resilient mindset are an indicator of good mental health and performance amongst first responders, despite being exposed to traumatic situations. However, very little research has been carried out on first responders in South Africa, making this study an important stepping stone towards gaining an understanding of the relationship between mindset as a resilience resource and perceived wellness of first responders in a South African context. Data were collected from 52 first responders using a structured questionnaire. The results indicate a statistically significant relationship between mindset and perceived wellness, with all the wellness factors indicating that the mindset of first responders plays a crucial role in their resilience and perception of wellness, necessitating additional research in this specialised field of disaster response.

## Introduction

Globally, there is an increase in the frequency and intensity of disasters and emergency situations, which has a major disruptive effect on affected societies (Centre for Research on the Epidemiology of Disasters 2021). This trend is emphasised in Africa, where conflict, malnutrition, poverty, diseases and social unrest are still some of the biggest factors contributing to societal vulnerability (Allison 2020; Cilliers 2007; Lancaster & Mulaudzi 2020). Developing countries such as South Africa are vulnerable to the impact of hazards, disaster situations and emergencies that strain the society’s ability to deal with these emergencies (Van Niekerk 2005). An overview of the term ‘disaster’ in the literature emphasises the fact that disasters happen because of the vulnerability of the society when one or several hazards overwhelm the capacity of the individual, group, society or environment to cope with or deal effectively with the impact of the hazard(s) (Everly & Lating 2017; Human Factor Combat Readiness 2020; International Federation of the Red Cross and Red Crescent Societies 2007; UNHCR 2009; United Nations Office of Disaster Risk Reduction 2015, 2019; Western Cape Local Government 2015; Zibulewsky 2001). Linked to the concept of vulnerability is coping or resilience, which refers to the ability of the affected individual, group, society or environment to find ways to deal with adversity, to learn from the adversity and to find ways to adapt to the adversity (Coetzee 2016). This ability to learn from and adapt to adversity is linked to the resources (and reserves of resources) with which the individual, group, society or environment must cope with the hazard (Graber, Pichon & Carabine 2015; Jaeschke 2016). Some of the resources, such as mindset (MS), appear to be causally linked to the willingness to be open to learn from previous experiences (Graber et al. 2015; Jaeschke 2016). Every community will have some capacity to deal with hazards or emergencies, but the level of vulnerability of a specific community would determine the capacity of the affected community to deal with the situation. From an emergency response perspective, the resources and capability to deal with a hazard would determine whether it is a routine emergency, a critical incident or a disaster (HFCR 2020). The necessity for effective disaster response is beyond dispute and forms an integral part of disaster risk reduction (DRR) strategies (DeFraia 2013; Fraher 2011). Members from emergency services, called first responders, play an important function by responding to emergencies and disasters in order to protect communities. As such, these members are often selected and trained to deal with the stressors involved with emergencies and disasters (Fraher 2011; Western Cape Local Government 2015).

However, regardless of how well-trained first responders are, they are humans who are exposed to stress that might affect their health and performance. Only in the last 20 years has some attention been given to the impact that disasters and emergencies have on first responders (Resnick et al. 2004). First responders are more exposed to work in different types of adverse situations and conditions that contain multiple stressors. These stressors often have a negative impact on their performance and ultimately their ability to respond to emergencies and disasters (Everly & Lating 2017; Fraher 2011; International Federation of the Red Cross and Red Crescent Societies 2011; O’Neil 2004; Sweeny, Matthews & Lester 2011). Understanding what types of stressors the adverse conditions entail and how it impacts first responders is important to counter the negative impact on performance. This concern was highlighted by the IFRC (2011), which stated that:

[*E*]ven when a response is well managed, emergencies are traumatic experiences. Being a volunteer does not make a person immune. First responders may also be victims, suffering the loss of loved ones or property or witnessing heart-wrenching situations. (p. 6)

Literature is replete with studies that have usually focused on the negative impact of disasters on first responders, a pathogenic approach that tends to highlight the prevalence of mental health issues and syndromes such as post-traumatic stress disorder (PTSD) and burnout (Arble & Arnetz 2019; Everly & Lating 2017; Fraher 2011; HFCR 2020; IFRC 2011; O’Neil 2004; Sweeny et al. 2011). Consequently, an overemphasis on the negative results of adversity leads to policy and treatments that address the problem one-dimensionally (HFCR 2020). However, as seen in an operational military environment, many people do not develop these syndromes but perform effectively and even thrive (Nindl et al. 2018; O’Neil 2002; O’Neil & O’Neil 2007; Sweeny et al. 2011; Van Wijk & Jarrad 2019). These findings in the military environment are echoed by findings on the general population, where 50% and more of people who are exposed to traumatic incidents display resilience (Bonanno et al. 2007; Bonanno & Mancini 2008). From a disaster response and societal resilience point of view, it is far more useful to address the performance of first responders multidimensionally by determining ways to strengthen and enhance the ability of first responders. To do so, it is necessary to determine the extent to which resilience factors such as MS contribute to the capacity to deal with adversity. However, within the South African context, very little research has been performed on this topic, making this study an important starting point (Ward, Lombard & Gwebushe 2006). This study therefore aims to determine what the link is between MS as a resilience resource and the perceived wellness (PW) of first responders. To do so, this study aimed to measure the MS and PW of first responders and also to explore the relationship between MS and PW of first responders. In addition, the study also aimed to explore the relationship between MS and perceived stress of first responders.

## Perceived wellness and resilience

Emergencies and disasters are by their very nature overwhelming. By definition, disasters overwhelm our capacity and resources to cope with a situation. This feeling of being overwhelmed can sometimes lead to feelings of disillusionment and helplessness, where first responders can even question their own ability to provide help to people affected by an emergency or disaster (Everly & Lating 2017; HFCR 2020). The unexpected and overwhelming nature of critical incidents, emergencies and disasters causes sudden changes to the physical situation, often creating massive damage to property, disrupting social support systems, leading to uncertainty and quite often causing serious injuries or death (Everly & Lating 2017; HFCR 2020; O’Neil 2002, 2004). This can be seen by the number of first responders who experience mental health problems (Everly & Lating 2017; Substance Abuse and Mental Health Services Administration 2018). This sentiment is echoed by international disaster relief organisations such as the United Nations and IFRC, where specific note is made of the need to strengthen and address the psychosocial needs of first responders (IFRC 2011).

### Resilience and wellness

Although there are indications that the stressors of working in a high-risk environment can potentially cause mental health problems, many people who choose to function in this environment perform very well without developing debilitating mental health problems (Nindl et al. 2018; O’Neil 2002; O’Neil & O’Neil 2007; Sweeny et al. 2011; Van Wijk & Jarrad 2019). One of the concepts that are used to describe this ability to perform effectively and thrive is called ‘resilience’. Maddi et al. (2007) described resilience as the capacity to respond adaptively to extreme stress. The ability to respond adaptively can also differ dramatically between settings and individuals. Therefore, there are significant differences between what different people would see as extreme stress and how to react to adapt to these stressors. Van Breda (2018) concurred and proposed that resilience should be studied in terms of (1) adverse conditions, (2) resilience as an outcome and (3) as a process. Therefore, resilience is the outcome or final state where the person has been able to recover from the adversity and be in a state or position similar to or better than what they were before the onset of the adverse conditions (Cilliers & Flotman 2016; Jaeschke 2016; Van Breda 2018; Van Wijk & Martin 2019). This description of resilience as an outcome is similar to the definition of ‘wellness’, where wellness is defined as the subjective perception of life quality of an individual as defined in terms of the mental, physical, spiritual, social, and environmental components (Cilliers & Flotman 2016; Gropp, Geldenhuys & Visser 2007). Gropp et al. (2007) further referred to wellness as a broad field of study that focuses on the quality of life and the general perception that people have that there will be positive outcomes to situations or circumstances. Wellness is further defined in terms of six dimensions, which include (1) self-acceptance, (2) social relationships with others, (3) personal growth (including creativity and pursuit of cognitive stimulating activities), (4) purpose, (5) autonomy and (6) environmental mastery, where environmental mastery includes financial wellness, personal health and safety and career development (Gropp et al. 2007). Wellness or resilience are therefore used interchangeably and refer to overall perceptions of life quality.

From a salutogenic approach, the question therefore is why some people continue to function effectively despite the adverse conditions experienced during disasters or emergencies. One possible answer to this question can be found in the way people strengthen and prepare themselves for possible adversity. To achieve the outcome of wellness or resilience, the individual or community needs to engage in a process of continuous learning from past adversity or mistakes and strengthening those factors or resources that would enable present or future performance in adversity. This process of preparation and strengthening is what Breda (2018) referred to as the resiliency process. Where resilience or wellness refers to the outcome, resiliency or the process of resiliency refers to the preparation and strengthening of resources that would facilitate effective functioning in adversity (Cilliers & Flotman 2016; Graber et al. 2015; Gropp et al. 2007; Jackson et al. 2003; Jaeschke 2016; Van Breda 2018). One of the factors that contribute to effective functioning in adverse conditions is the presence of factors or resources that assist, strengthen and enhance the ability of the individual, group or community to effectively deal with the adversity (Cilliers & Flotman 2016; Graber et al. 2015; Gropp et al. 2007; Jackson et al. 2003; Jaeschke 2016; Sisto et al. 2019; UNDRR 2015, 2019; Van Breda 2018; Wu et al. 2013). Bonanno et al. (2007), Bonanno and Mancini (2008) and Wu et al. (2013) identified and grouped factors or resources that promote resilience into three main heterogeneous groupings that include: (1) a number of person-centred resources (e.g. temperament and attitude, personality, coping strategies), (2) demographic factors (e.g. male gender, older age, greater education) and (3) sociocontextual factors (e.g. supportive relations).

Graber et al. (2015) and Jaeschke (2016) described resilience resources in terms of their protective function when dealing with adversity. In this protective function, resilience resources not only have an important role to promote adaptive resiliency processes, but also to mitigate the negative effects of adversity. Therefore, resilience resources refer to all resources or interventions at intrapersonal, interpersonal, spiritual, lifestyle, financial, physical and environmental levels that will enable the process of resiliency and ultimately wellness or resilience of the individual or community. Literature on resilience and resiliency all agree that adversity is a given and that at some stage in the life cycle of an individual or community, they will experience it in some form or the other (Cilliers & Flotman 2016; Graber et al. 2015; Gropp et al. 2007; Sisto et al. 2019; UNDRR 2015, 2019; Van Breda 2018). The presence of resilience resources does not take away adversity but rather enables the individual or community to deal more effectively with adversity in order to avoid some or all of the harmful effects thereof.

### Resilience resources

Resilience resources can be grouped into three main but overlapping groupings. These broadly correspond to the three categories that were identified by Bonanno et al. (2007) and Bonanno and Mancini (2008). *Internal resources* refer to those person-centred variables that are found at an intrapersonal level and enable the individual to deal with adversity (Bonanno et al. 2007; Bonanno & Mancini 2008; Van Breda 2018). This would include factors such as coping strategies and attitudes such as optimism or gratitude. In addition, it also includes internalised beliefs held by the individual about their own worth, competence and purpose and the extent to which they are open to new learning opportunities and whether they believe that difficulties are a normal part of life or view them as a threat. *Lifestyle resources* refer to those habitual patterns of behaviour that define an individual’s lifestyle (Hurley 2020; Logan-Greene et al. 2014). Lifestyle resources are linked to physical health and consist of factors such as getting sufficient sleep, healthy nutrition and sufficient exercise, although there is some overlap with internal resources. The final group or type of resources are *external resources*, which refer to the availability of strong and supportive social relationships (Van Breda 2018). These three types of resources, in combination and on their own, contribute to the building of resilience capacity of an individual and enhance the individual’s ability to deal with adversity. An example would be an individual who displays strong cognitive and problem-solving abilities (which in themselves are strong resources) who adds regular daily exercise to their routine. The physical benefits of exercise will not only enhance physical health and wellness but will also increase the individual’s cognitive ability and thereby increase overall resilience.

### Mindset as resilience resource

One of the resilience resources that is consistently identified as an important contributor to resiliency is the concept of ‘mental resilience’ or ‘mindset’ (Cilliers & Flotman 2016; Graber et al. 2015; Gropp et al. 2007; Sisto et al. 2019; Toseroni et al. 2015; Van Breda 2018; Van Wijk & Martin 2019). In literature, ‘mindset’ is described as an intricate combination of perceptions, coping strategies, dispositional attitudes and beliefs that an individual holds about difficulty, challenges, their own competence, learning opportunities and their ability to deal with difficulties. In summation the concept Mindset, as used in this study, is defined as the composition of cognitive states, emotional reactions, psychological attitudes and coping strategies that influence our perceptual appraisal of adversity and our ability to effectively deal with the situation leading to the implementation of actions and interventions that builds and strengthen resiliency (Bartone et al. 2008; Cohn & Pakenham 2008; Graber et al. 2015; O’Neil 2006; O’Neil & O’Neil 2007; O’Neil & Steyn 2007; Themanson & Rosen 2015; Tzur et al. 2016; Weisinger & Pawliw-Fry 2015).

The concept MS can be further described in terms of two subfactors, namely ‘focus’ and ‘attitude’ (Bierman & O’Neil 2019; Sisto et al. 2019; Weisinger & Pawliw-Fry 2015).

*Focus* refers to the dispositional attitudes in the manner in which individuals appraise or perceive a given situation (Bierman & O’Neil 2019; Jaeschke 2006; O’Neil 2006; O’Neil & O’Neil 2007; Sisto et al. 2019; Van Wijk & Martin 2019; Weisinger & Pawliw-Fry 2015). In the first place this refers to what individuals pay attention to when confronted with adversity. Is their focus on the positive or on the negative elements of the situation? Secondly, do the individuals focus only on what the problem or difficulty is, which leads to negative emotional states, or do they also focus on what to be grateful for, leading to positive emotional states? Lastly, is the focus of the individual on what they can control, or do they see themselves as being helpless? Can they make a positive appraisal of what they can control, or do they feel overwhelmed?

*Attitudes* refer to two basic elements: firstly, (1) the perception of the individual’s abilities to cope and secondly, (2) the individual’s perception of difficulties (Bartone et al. 2008; Cohn & Pakenham 2008; Graber et al. 2015; Gropp et al. 2007; O’Neil 2006; O’Neil & O’Neil 2007; Toseroni et al. 2015). Perception of one’s own abilities and perception of difficulties are often very intricately linked and influence each other. A positive perception of one’s own abilities often motivates an individual to seek out situations where they are challenged, as they see these opportunities to test their skills and to grow. This is also called a growth MS. The opposite is also true; someone with a negative perception of their ability to deal with adversity or difficulties would see adversity or difficulty as a threat to them, as this could potentially lead to failure. Failure would be unacceptable, as this would strengthen their underlying belief that they are not good enough. This fixed MS motivates people to only attempt tasks that they know how to achieve and be successful in, as this increases the probability of success. It also motivates these individuals to use avoidance as a strategy to ‘escape’ from possible failure. A growth MS motivates individuals to use active, action-orientated strategies to deal with difficulties.

Understanding the link between resilience, MS as a psychological resource that strengthens resilience and wellness will enable first responders to be empowered through awareness and skills training to deal more effectively with the adverse conditions that are characteristic of emergencies and disasters. In the next section, the methodology used in this study will be briefly discussed.

## Research methodology and design

This study followed a quantitative research approach as it involved the identification, explanation and prediction of relationships between two or more variables (Bergh & Theron 2006; Blanche, Durrheim & Painter 2006; Fouche et al. 2011; Leedy 1997). The purpose of this study was to determine the relationship between two variables, MS as a resilience resource and PW. In addition, the relationship between MS and perceived level of stress related to the function of first responders was investigated.

Firstly, a detailed literature review was conducted where the key concepts of *resilience resources, resilience, disaster, hazards, emergencies, adversity* and *first responders* were investigated and described. Sources that were used included textbooks, peer-reviewed articles, training manuals, unpublished dissertations, governmental publications and Internet articles.

Empirical data were collected using objective and mechanical measures that assisted with the quantification of responses (Bergh & Theron 2006; Bernstein et al. 2005; Struwig & Stead 2001). A structured questionnaire consisting of 5 sections and 115 questions that measured resilience resources and perceptions of wellness (perceived wellness and resilience resources questionnaire [PWRRQ]) was used specifically for the purpose of this study. This questionnaire was designed by a panel of psychologists based on the literature and measures perceptions of stress experienced, perceptions of wellness and measures resilience resources. After the instrument was developed, two pilot studies were carried out on samples within the South African National Defence Force (SANDF).

The PWRRQ was administered to first responders using different methods of administration, including Google Forms and paper-and-pencil based questionnaires, depending on the method best suited to the participant. The sample that was used was a ‘convenience sample’. This means that respondents were selected based on availability and willingness to participate (Blanche et al. 2006). A minimum number of 25 respondents were needed to participate in the study to enable the use of Pearson and Spearman correlational methods (Bonnet & Wright 2000; De Winter, Gosling & Potter 2016). A total of 52 respondents participated in the end, making this the final sample size (*N* = 52), which consisted of first responders from security companies, South African Police Service (SAPS) members, Search and Rescue, Fire and Rescue services and Emergency Medical Services (EMS) personnel in the Gauteng Province of South Africa who were willing to participate in this study.

The results were captured using electronic programmes such as Excel or Access. The data were analysed by means of descriptive statistics (correlations) using the JASP 0.14.10 statistical programme. Correlations provided an indication of the degree of relationship between two or more variables and were therefore most suitable to answer the research aim and objectives of this study (Bergh & Theron 2006; Bernstein et al. 2005; Struwig & Stead 2001).

As this study involved human respondents, it was important in the design to include measures that would ensure the safety and psychological comfort experienced by the participants to a level that is similar to what would be experienced as part of their daily lives. To achieve this, possible respondents received an information letter explaining the aim and purpose of the study, their role (should they choose to participate), voluntary participation and exit, rewards and feedback, anonymity and confidentiality.

Although the study focused on perceptions, attitudes and lifestyle descriptions, it did not tap into psychologically sensitive aspects such as descriptions of previous traumatic experiences. In addition, the respondents were offered the opportunity to see a psychologist for debriefing should they wish to do so. Each respondent received a feedback report, but no reward for participation in the study was offered. Ethical clearance was received from the North-West University Faculty of Natural and Agricultural Sciences Research Ethics Committee (FNAS-REC), with the ethics number NWU-01264-21-A9.

### Ethical considerations

Based on approval by the Faculty of Natural and Agricultural Sciences Research Ethics Committee (FNAS-REC), the Faculty of Natural and Agricultural Sciences Ethics Committee approved this study. The North-West University Senate Committee for Research Ethics (NWU-SCRE) granted its permission for the study to be initiated, using the given ethics number NWU-01264-21-A9.

## Results

This section will be used to describe the results of the study. The first section will specifically focus on the overall wellness (OW) and a description of the elements of PW. In addition, the perceived level and frequency of stress will be described. The second part of this section will focus on a description of the correlations between the main concepts that were measured. Correlations were carried out for Mindset (MS) and OW, MS and the subcategories of OW and finally OW and the MS subfactors.

## Perceived wellness

**FIGURE 1:**
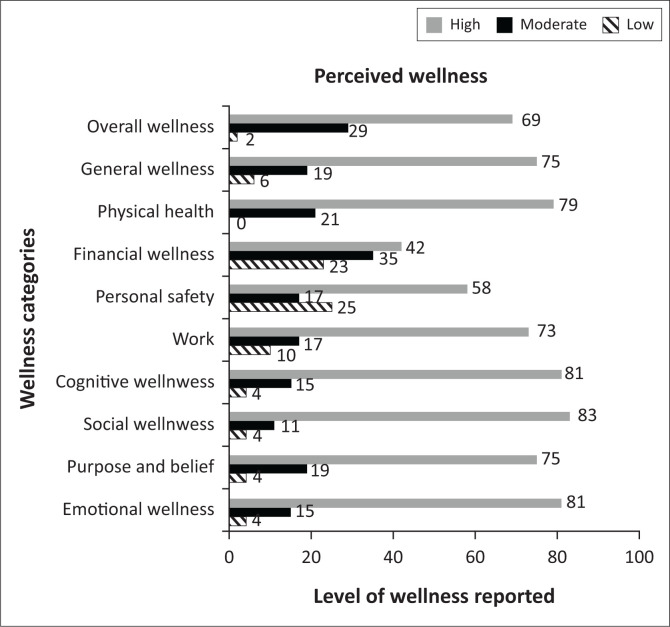
Perceived wellness.

Overall wellness provides an indication of the combined results of the subfactors (general wellness, physical health, financial wellness, personal safety, work satisfaction, cognitive wellness, social wellness, purpose and belief and emotional wellness). Figure 1 illustrates the perception of wellness as reported by the respondents completing the PWRRQ. Emotional wellness was indicated by 81% of the respondents to be high, with 15% reporting moderate levels and 4% indicating low levels of emotional wellness. Seventy-five per cent of the respondents indicated that they experience high levels of purpose and beliefs, with 19% reporting moderate and 4% low levels of purpose and beliefs.

The majority of respondents (83%) indicated high levels of wellness in terms of their social connectedness, whilst 11% indicated moderate levels and 4% experienced low levels of social wellness. Cognitive wellness was reported as high by 81% of the respondents, with 15% reporting moderate and 4% low levels of cognitive wellness.

These relatively high levels of wellness were also reported for work wellness (73%), physical health (79%) and general wellness (75%). However, it seems that there were some concerns regarding personal safety and financial wellness. Under financial wellness, 58% of the respondents reported moderate to low levels of satisfaction. A similar trend can be seen under personal safety, where 42% of the respondents reported moderate to low levels of satisfaction. Although the OW remained high for the majority of the respondents, these two subfactors did have a negative impact on the overall perception of wellness.

It is clear that the majority (69%) of the respondents demonstrated that they experience high levels of OW (the combined results of the subfactors, for example, general wellness, physical health, financial wellness, personal safety, work satisfaction, cognitive wellness, social wellness, purpose and belief and emotional wellness), whilst 29% reported moderate levels of OW and only 2% experienced low levels of OW. This is an indication that the largest part of the sample experiences moderate to high levels of PW. This means that the largest part of the sample has a subjective perception of a high life quality in terms of the mental, physical, spiritual, social and environmental components of their life. The second variable that was studied in this research project was MS as one of the resilience resources. The findings for MS will be discussed in the next section.

## Resilience resources, mindset and first responders

In this section, the MS of the first responders who participated in this study will be discussed. In addition, the two subfactors of MS, namely *focus* and *attitude* will also be discussed.

**FIGURE 2:**
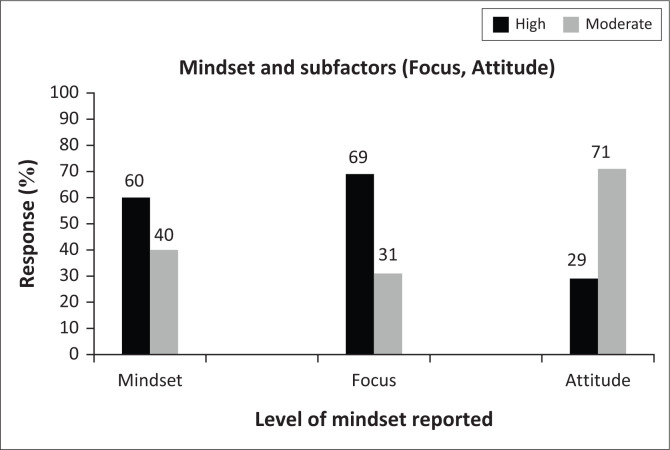
First responder mindset.

Figure 2 demonstrates that the majority of respondents or 60% who participated in this study reported high levels of MS. The remaining 40% reported moderate levels of MS. What was also found in this study was that 69% of the first responders who participated in this study achieved high levels of positive focus, whilst the remaining 31% achieved moderate levels of positive focus. Overall, the majority of respondents therefore displayed a dispositional focus on optimism, gratitude and hope, commitment to a purpose and beliefs and confidence in their own abilities – all factors that seem to contribute to a MS of resilience (Bartone et al. 2008; Graber et al. 2015; Gropp et al. 2007; Steadman 2011; Steinberg & Kornguth 2009; Van Wijk & Martin 2019; Weisinger & Pawliw-Fry 2015).

Furthermore, the majority (71%) of first responders achieved moderate attitude levels, whilst only 29% achieved high levels. None of the sample achieved low levels. Although the overall subfactor was relatively high, it was interesting that it was markedly lower than the scores achieved on the subfactor focus. The link between perceptual appraisal of the adversity versus perceptual appraisal of own abilities was confirmed by the literature, where it was found that the manner in which an individual deals with difficulties predicts the extent to which they will experience distress or negative stress (Cohn & Pakenham 2008; O’Neil 2006; O’Neil & O’Neil 2007; O’Neil & Steyn 2007; Weisinger & Pawliw-Fry 2015). Those individuals who rely on emotion-focused ‘avoidance’ strategies tend to experience greater distress and tended to see difficulties as threats. Individuals who experienced difficulties as opportunities for learning and growth tended to use problem-focused strategies. One possible conclusion could be that the majority of first responders who participated in this study seem to view difficulties as threats to their own self-concept, and therefore they tend to view difficulties as obstacles that either require avoidance or need to be completed as quickly as possible without making any mistakes. Either way, this attitude will lead to increased stress levels.

## Perceived stress

**FIGURE 3:**
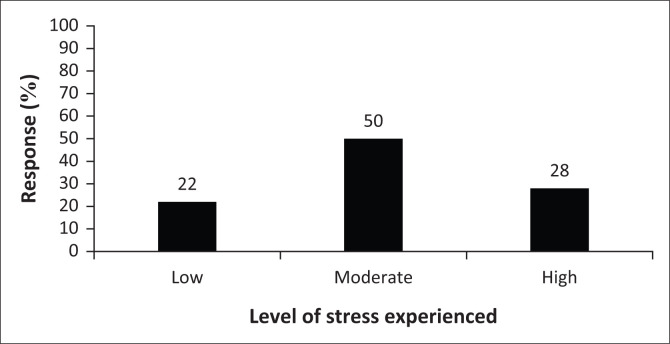
Level of stress experienced.

When completing the PWRRQ, the respondents had to indicate the level of stress that they experienced as first responders. As seen in Figure 3, there seemed to be an almost normal distribution between the reported low stress levels (22%), moderate stress levels (50%) and high stress levels (28%). Therefore, at least 78% of the sample reported moderate to high stress levels, which would be understandable given the type of work environment in which they function. This finding is congruent with findings in literature that highlight that first responders are a ‘high-risk’ grouping because of their continued exposure to stressors in their work environment (Everly & Lating 2017; IFRC 2011; Pietrantoni & Prati 2008).

As seen in Table 1, there is a moderate and statistically significant relationship between the perceived level of stress and the frequency of stress experienced by first responders in this study. In addition, there is a moderate, statistically significant negative relationship between perceived level of stress and the MS of the participants in this study. The implication of this is that stress levels are inversely related to the MS of the first responder. The stronger the MS of the first responder, the more likely they are to perceive lower levels of stress.

**TABLE 1:** Perceived level and frequency of stress and mindset.

Variable 1	Variable 2	Pearson		Spearman	
		*r*	*p*	rho	*p*
What level of stress do you experience?	How frequently do you experience stress?	0.508***	< 0.001	0.514***	< 0.001
What level of stress do you experience?	Mindset	−0.400**	0.004	−0.423**	0.002
How frequently do you experience stress?	Mindset	−0.302*	0.033	−0.335*	0.017

* , *p* < 0.05;

** , *p* < 0.01;

*** , *p* < 0.001.

## Correlations between mindset and overall wellness

In this section, the results of the correlations that were performed using the JASP 0.14.10 programme between MS and OW and between MS and the subcategories of PW will be discussed. In all the tables, the results for both the Pearson and Spearman tests of correlation will be indicated.

Table 2 provides the correlation between the main variables that were investigated in this study and confirms the primary hypothesis that a positive relationship exists between the concepts MS and PW.

**TABLE 2:** Mindset, mindset subfactors and perceived wellness.

Variable	Overall wellness
**1. Focus**	
Pearson’s *r*	0.571***
*p*-value	< 0.001
Spearman’s rho	0.509***
*p*-value	< 0.001
**2. Attitude**	
Pearson’s *r*	0.471***
*p*-value	< 0.001
Spearman’s rho	0.440**
*p*-value	0.001
**3. Mindset**	
Pearson’s *r*	0.629***
*p*-value	< 0.001
Spearman’s rho	0.593***
*p*-value	< 0.001

* , *p* < 0.05;

** , *p* < 0.01;

*** , *p* < 0.001.

As is seen in Table 2, there is a moderate to strong positive relationship between MS and PW that is statistically significant, thereby confirming one of the research objectives of this study. When the MS subfactors, focus and attitude, were correlated with OW (Table 2), it is clear that statistically significant relationships were found that ranged from 0.47 for attitude to 0.57 for focus.

In Table 3, MS is shown to have positive, statistically significant relationships with ranges between 0.36 (work wellness) and 0.53 (social wellness) with all of the wellness factors that were measured in this study.

**TABLE 3:** Mindset and wellness factors.

Variable	Mindset
**1. Emotional wellness**	
Pearson’s *r*	0.495***
*p*-value	< 0.001
Spearman’s rho	0.478***
*p*-value	< 0.001
**2. Cognitive wellness**	
Pearson’s *r*	0.464***
*p*-value	< 0.001
Spearman’s rho	0.424**
*p*-value	0.002
**3. Work wellness**	
Pearson’s *r*	0.361**
*p*-value	0.010
Spearman’s rho	0.296*
*p*-value	0.037
**4. Purpose and belief**	
Pearson’s *r*	0.473***
*p*-value	< 0.001
Spearman’s rho	0.510***
*p*-value	< 0.001
**5. Physical health**	
Pearson’s *r*	0.400**
*p*-value	0.004
Spearman’s rho	0.401**
*p*-value	0.004
**6. Social wellness**	
Pearson’s *r*	0.530***
*p*-value	< 0.001
Spearman’s rho	0.503***
*p*-value	< 0.001
**7. Personal safety**	
Pearson’s *r*	0.460***
*p*-value	< 0.001
Spearman’s rho	0.428**
*p*-value	0.002
**8. Financial wellness**	
Pearson’s *r*	0.376**
*p*-value	0.007
Spearman’s rho	0.338*
*p*-value	0.016
**9. General wellness**	
Pearson’s *r*	0.503***
*p*-value	< 0.001
Spearman’s rho	0.494***
*p*-value	< 0.001

* , *p* < 0.05;

** , *p* < 0.01;

*** , *p* < 0.001.

These results clearly indicate that there is a statistically significant relationship between MS and PW, including all the subfactors of both variables. The implications and recommendations for further research will be discussed in the next section.

## Discussion

The results of this study indicate that MS and the subfactors focus and attitude have a statistically significant relationship with PW and with all the wellness factors. These findings are confirmed by findings of other studies that werecarried out with first responders in adverse situations, where the components of MS have been shown to be highly predictive of positive coping behaviour during adversity (Bartone et al. 2008; Crum et al. 2017; Dowdall-Thomae et al. 2012; Weisinger & Pawliw-Fry 2015). Themansonand Rosen (2015) indicated that a MS of self-efficacyis related with improved performance and that a resilient MS contributes to positive management of extended periods of stress, trauma and adversity (Bonanno et al. 2007).The relationships that were found between the subfactorsof MS are further supported by Bartone et al. (2008),Wu et al. (2013) and Jamieson et al. (2018) that emphasise the finding that a MS where the individual perceives themselves to be in control (as opposed to being helpless) and having sufficient resources contributes positively to resilience and performance during adversity.

The implications are that MS as a concept is applicable to first responders in the South African context and that further research in this field is necessary. The field of disaster psychology or psychology within the field of Disaster Risk Management (DRM) is sadly neglected, and the findings of this study indicate the necessity for further research.

The aim of this study was to determine the relationship between MS as one of the resilience resources and PW of first responders. This was achieved with the results of this study, indicating that MS and the subfactors focus and attitude have a statistically significant relationship with PW as well as with all the wellness factors. The objectives that were set for this study (1) to measure MS of first responders, (2) to measure PW of first responders and (3) to explore the relationship between MS and PW of first responders was achieved and the results were discussed.

The methodological design for this study was sound and the instrument that was used to gather the data was theoretically grounded and displayed statistical robustness in measuring perceptions of stress levels experienced, perceptions of wellness and resilience resources. The study paves the way for additional, in-depth research to not only further explore this topic but also to develop disaster or operational psychology as an academic and applied field of disaster management in South Africa.

Although the findings are relevant and valid for the specific context, the sample size, composition and sampling methodology were insufficient for the results to be generalised to whole population of first responders in South Africa. A larger and more diverse sample, chosen using random sampling methods, would improve the generalisability of the results. This study only established that there is a statistically significant relationship between the variables and subvariables. Bigger samples could influence this result and, most probably, based on theory and literature, indicate stronger relationships. It does not provide any further insight into how variables such as MS can be developed, trained or enhanced within the first responder environment. Furthermore, this study might indicate a relationship between the variables, but it neither indicates the impact of stress, nor the stressors involved. Once these factors have been researched in more depth, it would form the basis for the development of training and support programmes that would assist with the selection, training and development of first responders. Additional research in this specialised field of disaster response is needed.

## Conclusion

The purpose of this study was to give an overview of the concept ‘mindset’ as a resilience resource and the conceptual link with wellness of first responders in the Gauteng province of South Africa. There is sufficient evidence in the literature proving that first responders are exposed to a wide range of work-related stressors and levels of adversity. Furthermore, there are wide-ranging studies that make a link between wellness, performance and resilience and the use of resilience resources in the development and enhancement of wellness. One of the resilience resources that consistently contributes to wellness, resilience and consistent performance is that of psychological mindset, a coping mindset or resilient mindset. In the international context, a coping or resilient mindset seems to be an indicator of good mental health and performance amongst first responders, despite being exposed to traumatic situations and work-related adversity. However, very little research has been carried out on the situation of first responders in South Africa or more specifically in Gauteng, therefore making this study an important stepping stone towards gaining an understanding of this relevant topic. This study measured the perceptions of stress, wellness and resilience resources of 52 first responders using the PWRRQ. The findings of this study indicate that mindset and the subfactors focus and attitude have a statistically significant relationship with PW and the wellness factors. This finding is substantiated by the literature and therefore, despite various limitations, opens the way for more in-depth research in this specialised field of disaster response that would form the basis for the development of training and support programmes that would assist with the selection, training and development of first responders.
